# Slow Accumulations of Neural Activities in Multiple Cortical Regions Precede Self-Initiation of Movement: An Event-Related fMRI Study

**DOI:** 10.1523/ENEURO.0183-17.2017

**Published:** 2017-10-30

**Authors:** Honami Sakata, Kosuke Itoh, Yuji Suzuki, Katsuki Nakamura, Masaki Watanabe, Hironaka Igarashi, Tsutomu Nakada

**Affiliations:** 1 Center for Integrated Human Brain Science, Brain Research Institute, University of Niigata, Niigata 951-8585, Japan; 2 Primate Research Institute, Kyoto University, Inuyama City, Aichi 484-8506, Japan

**Keywords:** free will, intention, decision making, self-initiated movement, Bereitschaftspotential

## Abstract

The neural processes underlying self-initiated behavior (behavior that is initiated without an external stimulus trigger) are not well understood. This event-related fMRI study investigated the neural origins of self-initiated behaviors in humans, by identifying brain regions that increased in neural activities several seconds prior to self-initiated movements. Subjects performed a hand grasping task under two conditions: a free-timing and cued timing condition. The supplementary motor area (SMA) began to activate several seconds prior to self-initiated movement (accounting for hemodynamic delay), representing a potential blood oxygenation level-dependent (BOLD) signal correlate of the readiness potential (RP) on electroencephalogram (EEG), referred to here as “readiness BOLD signals.” Significant readiness BOLD signals were also observed in the right frontoparietal areas, precuneus, and insula, all of which are known to contribute to internally-generated behaviors, but with no prior evidence for such early and slow accumulation of neural activities. Moreover, visual and auditory cortices also exhibited clear readiness BOLD signals with similar early onsets, even absent external stimulation. Slow accumulation of neural activities throughout distributed cortical areas, including sensory, association, and motor cortices, underlies the generation of self-initiated behaviors. These findings warrant reconsideration of the prevailing view that the SMA or some other specific locus in frontoparietal cortex serves as the ultimate neural origin of self-initiated movement.

## Significance Statement

A stimulus can trigger a chain of neural activities that culminate in a behavior, but behaviors can also be initiated endogenously, without an external stimulus. We investigated the neural origins of self-initiated behaviors by identifying brain regions that displayed increased neural activity several seconds before onset of self-initiated movements. Our analysis revealed slow accumulation of neural activities that preceded self-initiated movements in several brain regions including the sensory, association, and motor cortices. We propose that endogenous accumulation of neural activities in networks of multiple cortical regions underlie generation of self-initiated movement.

## Introduction

In the classical view of human behavior that underlie behaviorism or stimulus-response theory (Skinner, 1953; Pavlov and Anrep, 2003), a stimulus input triggers a chain of neural activities in the brain that culminate in a behavior output. However, behaviors can also be initiated endogenously, absent an external stimulus. The neural mechanisms responsible for endogenously initiated behaviors are not well understood. Recently, an evidence accumulator model of perceptual decision-making identified spontaneous neural firing in the brain as the cause of internally initiated behaviors (Schurger et al., 2012, 2016; Bode et al., 2014). In the original accumulator model of perception, external sensory information, called “evidence,” is integrated over time in the brain, and when the firing rate of neurons reaches a threshold, a perceptual decision is made (Usher and McClelland, 2001; Smith and Ratcliff, 2004; Gold and Shadlen, 2007; Heekeren et al., 2008). When applied to internally generated actions, stochastic neural activities that occur spontaneously in the absence of stimulus inputs are accumulated over time until it reaches a threshold, at which point a behavior occurs (Schurger et al., 2012, 2016; Bode et al., 2014). The model has successfully explained behavioral and electrophysiological data recorded from subjects performing the Libet’s paradigm (Libet, 1985), in which they were instructed to press a button whenever they spontaneously “felt the urge” to do so (Schurger et al., 2012).

We have yet to identify the neural substrates of the accumulator, or the brain loci where neural activities accumulate. Stochastic neural activities are ubiquitous in the brain, and the accumulator model does not make specific predictions about where evidence accumulates, absent external inputs. The traditional view was that the supplementary motor area (SMA) represents the neural origin of endogenously generated actions (Eccles, 1982). That is, accumulated neural activities in SMA initiate a chain of neural events in other motor-related areas that culminate in behavior (Jenkins et al., 2000; Schurger et al., 2012; Schultze-Kraft et al., 2016). This hypothesis is based on observations of readiness potential (RP) on electroencephalogram (EEG), which is a gradual buildup of negative potential beginning up to one second or more before self-initiated movement (Kornhuber and Deecke, 1965). The early phase of RP originates in SMA (Shibasaki and Hallett, 2006). Functional magnetic resonance imaging (fMRI) studies confirmed that ramping activation in the SMA precedes behavior during free-decision tasks (Weilke et al., 2001; Cunnington et al., 2002; Soon et al., 2008, 2013), which represents a potential blood oxygenation level-dependent (BOLD) signal correlate of RP, referred to here as the “readiness BOLD signal.”

It is unknown if this buildup of neural activities occurs only in the SMA, or also in other brain regions. Spatial patterns of fMRI activation in the parietal and fontal cortex contain information about decisions that the subject has not yet consciously made, suggesting that the neural precursors of motor decisions originate in higher-order cortices outside the SMA (Soon et al., 2008). Even earlier in the stream of information processing, resting state neural activities in the sensory cortex can be functionally connected with regions in frontoparietal and sensorimotor cortices (Wang et al., 2008), and influence behavior (Bengson et al., 2014). It is theoretically possible that any region along the full pathway of information processing, from the sensory cortex via the association areas to the motor cortex, can serve a neural substrate for evidence accumulation.

**Figure 1.**
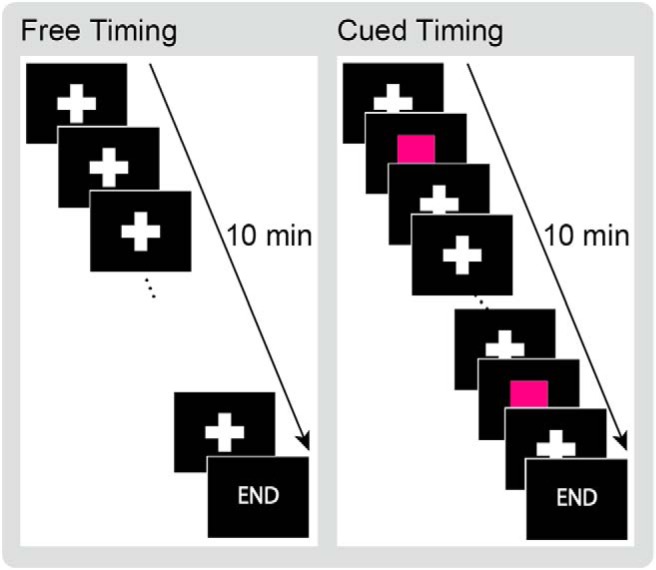
Task design. In the free-timing condition, each subject freely decided when to move his right hand while fixating his gaze on a stationary mark. In the cued timing condition, the subject produced movements in response to a visual cue.

We investigated this hypothesis by identifying brain regions with slow buildups of neural activities during the premovement period, as indexed by readiness BOLD signals. Regions of interest (ROIs) were broadly defined as brain regions that have been shown or implicated to be involved in various versions of self-initiation/free-decision tasks. Although previous fMRI experiments have not found readiness BOLD signals in areas other than the SMA (Soon et al., 2008, 2013), this was possibly due to an experimental condition inherent to the classic Libet’s paradigm (Libet, 1985). That is, the use of rapidly updating visual stimuli, to mark the time of subjective decision, was not an ideal method for studying spontaneous neural activities absent sensory inputs, particularly within the sensory cortices. In the present experiment, subjects moved their right hand when they felt the spontaneous urge to do so, while fixating a cross mark that was unchanged for the entire duration of fMRI recording (Fig. 1). For control, they performed the task cued by a visual stimulus. The fMRI signals were back-averaged time locked to the movement, to identify brain regions that began to activate before movement, specifically in the free-timing task.

## Materials and Methods

### Subjects

Twenty paid volunteers with normal or corrected-to-normal visual acuity, all healthy and right-handed, participated in this study (mean age: 22.3 years, range: 18-34 years; all males). The fact that all subjects were males was a limitation of this study, although sex differences have not been reported for neural correlates of self-initiated movement (Cunnington et al., 2005; Shibasaki and Hallett, 2006; Bode et al., 2014). Human subjects were recruited at the University of Niigata. Written informed consent was obtained from all subjects, and this study was approved by the Internal Review Board of the University of Niigata and by the Human Research Ethics Committee of the Primate Research Institute, Kyoto University.

### Behavioral task

Each subject performed a hand grasping task with his right hand in two conditions: a free-timing condition and a cued timing condition (Fig. 1). In the free-timing condition, the subject squeezed a MRI-compatible ball-shaped response device (Current Designs, HHSC-2x1-PNE) with his right hand when he felt the urge to do. Throughout the condition the subject fixated his eyes on a cross mark placed at the center of a screen. Subjects were instructed to make the squeezing movement at a pace of approximately two or three times per minute; however, they were also instructed to never to count the time. In the cued timing condition, each subject performed the squeezing movement in response to a visual stimulus (red square), presented at the same position as the central cross mark that the subjects were instructed to fixate (Fig. 1). The cue stimulus disappeared immediately after the response. The timing of cue presentation during the cued timing condition was temporarily matched to the subject’s own spontaneous movements recorded during the free-timing condition, which was always performed first. Therefore, the number and timing of responses were matched between the two conditions. Each task lasted 10 min 30 s, and data collected during the first 30 s were discarded. Eighteen subjects completed the experiment by performing each task twice. Due to time constraint, one subject performed the free-timing task twice and cued timing task once, and another subject performed each task once. Stimulus presentation and response acquisition were controlled by a MATLAB (MathWorks) script, using the Psychophysics Toolbox extensions (Brainard, 1997; Pelli, 1997; Kleiner et al., 2007).

### Image acquisition

A Signa LX 3.0-Tesla (GE Medical System) imaging system was used for all imaging. The functional images were obtained using an 8-channel head coil and an interleaved multi-slice gradient-echo echo-planar pulse sequence (TR, 1000 ms; field of view, 200 × 200 mm; matrix, 64 × 64; TE, 30 ms; flip angle, 70°; slice thickness, 5 mm; slice spacing, 2.5 mm). Fifteen axial slices covered the whole cerebrum. The short repetition time for fast temporal sampling led to a compromised spatial resolution in the inferior-superior dimension. The TR of 1 s was shorter than those used in other relevant studies in the literature (Cunnington et al., 2002; Lau et al., 2004; Soon et al., 2008, 2013), giving us a unique advantage in analyzing the time course of BOLD signals. A low spatial resolution was consistent with, and relatively unproblematic for, our ROI-based analysis, which inherently had a coarse spatial resolution. Each fMRI scan lasted 10 min 30 s, and the data for the first 30 s were discarded to ensure a steady state.

### Data analysis

In the preprocessing step, functional images were realigned to the first image in the series to correct for within-scan head motions, coregistered with the T1-weighted structural image for each subject, normalized to the MNI space, and spatially smoothed by a 8-mm full-width at half-maximum Gaussian kernel, using Statistical Parametric Mapping 12 software (SPM12, Wellcome Department of Cognitive Neurology, United Kingdom). Then the data were transformed to the unit of percentage signal change, where the baseline was defined as the average of the entire 10 min-long signal.

**Figure 2.**
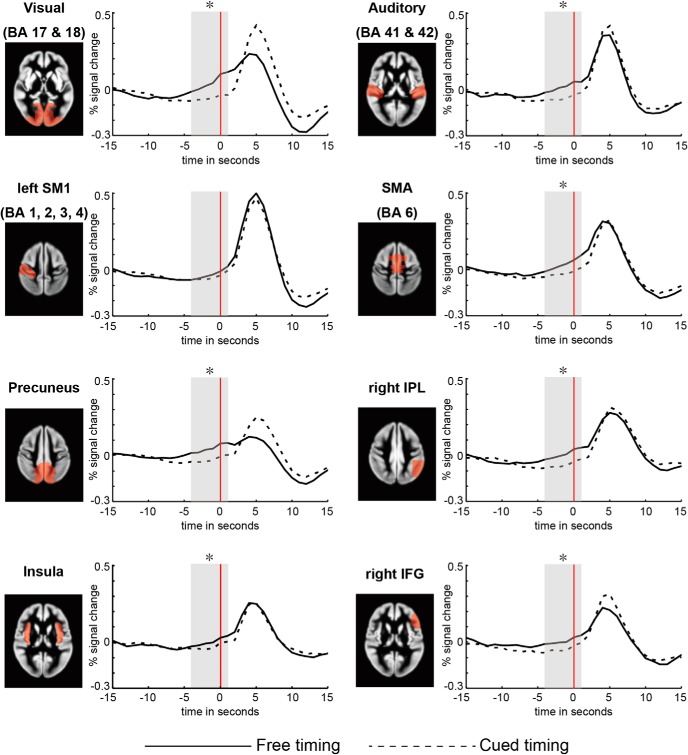
fMRI signal time courses. The red line represents the time of movement onset, defined as *T* = 0 (s). The shaded region indicates the time window (−4 ≤ *T* ≤ 1) in which the fMRI responses in the free-timing and cued-timing conditions were compared, and asterisks indicate statistically significant differences (*p* < 0.05). Even absent an external stimulus, significant buildups of activation during the premovement period were observed in the visual cortex, auditory cortex, SMA, precuneus, right IPL, right IFG, and insula.

We searched for an evidence of readiness BOLD signals in predefined ROIs, which were broadly defined to include any brain area that has been shown or suggested to be involved in performing various types of self-initiation and free-decision tasks (Jahanshahi et al., 1995; Jenkins et al., 2000; Cunnington et al., 2002; Lau et al., 2004; Soon et al., 2008; Desmurget et al., 2009; Fried et al., 2011; Haggard, 2008; Hoffstaedter et al., 2013): primary sensorimotor area (SM1, BA 1, 2, 3, 4), SMA (medial part of BA6), anterior cingulate cortex (ACC), inferior parietal lobule (IPL), middle frontal gyrus (MFG; including a part of premotor cortex), basal ganglia, insula, inferior frontal gyrus (IFG; including a part of premotor cortex), superior parietal lobule (SPL), frontopolar cortex (BA 10), posterior cingulate cortex (PCC), and precuneus. Visual (BA 17 and 18) and auditory (BA 41 and 42) sensory cortices were also included, because they might play a role in the self-initiation of movement, as argued above. The left and right hemispheres were distinguished for regions that have been reported to show functional hemispheric asymmetry during self-initiated movement: SM1, IPL, SPL, MFG, and IFG (Jahanshahi et al., 1995; Jenkins et al., 2000; Lau et al., 2004; Hoffstaedter et al., 2013). The ROIs were specified using WFU_Pickatlas (Maldjian et al., 2003, 2004). After extracting ROI data using MarsBar (http://marsbar.sourceforge.net/), the data were segmented from 15 s before and 15 s following the onset of each hand movement. Clipped epochs at the beginning and the end of the recording were not used. Next the data were averaged time locked to movement onset across subjects to obtain event-related fMRI responses for each ROI and task (Fig. 2). Finally, we performed paired *t*-tests with a significance threshold of *p* = 0.05 (one tailed) to test a hypothesis that, in the time window of 4 s before and 1 s following movement onset (defined as *T* = 0), there were increases in activation in the free-timing task compared to the cued timing task. This time window (−4 ≤ *T* ≤ 1) was clearly earlier than, and had small overlap with, the fMRI response associated with movement execution, which peaked at 5 s after movement due to hemodynamic delay (Fig. 2). All *p* values were corrected for multiple comparisons by the Benjamini and Hochberg (Benjamini and Hochberg, 1995) false discovery rate (FDR) method, unless otherwise noted.

### Data visualization

Separate from the statistical analysis described above, whole-brain activation maps were created for an intuitive visualization of brain activities that occurred during the premovement period. Preprocessed data were segmented using the time window of −15 ≤ *T* ≤ 0. Segments were averaged time locked to the onset of movement for each subject and each task. We then performed paired *t*-tests between the free- and cued-timing conditions for each time point and each voxel to obtain a series of uncorrected *t*-maps representing the contrast of “free-timing condition” minus “cued timing condition.” These analyses were performed using an original MATLAB script. Finally, *t*-maps were overlaid on an MNI template brain using FSLeyes (FSL image viewer; Smith et al., 2004; Jenkinson et al., 2012).

## Results

### Behavior

In the free-timing condition (and also in the cued timing condition), subjects made hand-grasping movements at an average pace of once every 31.1 (±7.5 SD) s. In the “cued timing” condition, the mean reaction time was 0.50 (±0.11 SD) s, and there were no missed trials in any subject.

### fMRI

Event-related fMRI signals for each ROI are plotted in Figure 2. Due to hemodynamic delay, fMRI signal changes associated with movement peaked around 5 s after movement onset. The amplitudes of these signal changes were comparable between the free-timing and cued-timing conditions in the left SM1 (*t* = 1.04, *p* > 0.05, 4–6 s). This result was expected and it confirmed that the motor component of the task was matched between the two conditions.

Readiness BOLD signals were observed in several ROIs, including (but not limited to) the SMA (Fig. 2). In the SMA, activation in the free-timing condition was significantly stronger than that in the cued timing condition during the premovement time window [*t*_(19)_ = 2.73, *p* = 0.036], which was consistent with previous findings. In addition, significant readiness BOLD signals were also observed in the right IPL [*t*_(19)_ = 2.75, *p* = 0.036], precuneus [*t*_(19)_ = 2.67, *p* = 0.036], right IFG [*t*_(19)_ = 2.29, *p* < 0.050], insula [*t*_(19)_ = 2.25, *p* < 0.050], visual cortex [*t*_(19)_ = 4.07, *p* < 0.01], and auditory cortex [*t*_(19)_ = 2.55, *p* = 0.038]. The readiness BOLD signals in these regions commenced around five seconds before movement onset (*T* = −5), while the BOLD response in the SM1 peaked at five seconds after movement (*T* = 5). In other words, the neural activities underlying readiness BOLD signals began approximately ten seconds before movement execution, accounting for the hemodynamic delay.

Evidence for readiness BOLD signals were weak or absent in the other ROIs: right MFG [*t*_(19)_ = 1.84, *p* = 0.091], SPL [left hemisphere, *t*_(19)_ = 1.77, *p* = 0.091; right hemisphere, *t*_(19)_ = 1.75, *p* = 0.091], SM1 [left hemisphere, *t*_(19)_ = 0.65, *p* = 0.292; right hemisphere, *t*_(19)_ = 1.36, *p* = 0.154], ACC [*t*_(19)_ = 0.12, *p* = 0.452], left IPL [*t*_(19)_ = 1.34, *p* = 0.154], left MFG [*t*_(19)_ = 0.90, *p* = 0.266], basal ganglia [*t*_(19)_ = 0.46, *p* = 0.342], left IFG [*t*_(19)_ = 0.69, *p* = 0.292], frontopolar cortex [*t*_(19)_ = 0.88, *p* = 0.266], and PCC [*t*_(19)_ = 0.77, *p* = 0.287].

**Figure 3.**
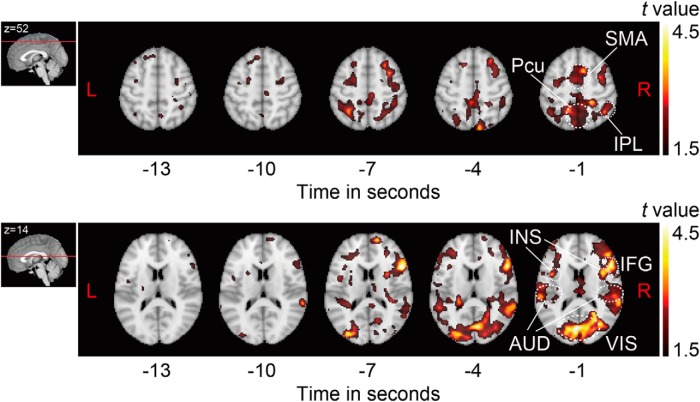
Subtraction *t*-maps during premovement period. Subtraction *t*-maps (free timing minus cued timing) showed neural activities in multiple cortical areas, beginning several seconds before the onset of self-initiated movement, defined as *T* = 0. AUD, auditory cortex; INS, insula; Pcu, precuneus; VIS, visual cortex; L, left; R, right.

For the purpose of visualization, Figure 3 provides snapshots of brain activation at several time points before movement (*T* = −13, −10, −7, −4, −1). A slow increase in neural activities was observed in the SMA, right IPL, precuneus, right IFG, insula, visual cortex, and auditory cortex, consistent with the results of ROI analysis.

## Discussion

Since the discovery of RP (Kornhuber and Deecke, 1965), and Libet’s experiment on “free will” (Libet, 1985), the SMA has long been considered the most important, and possibly the only, site of neural origin for self-initiated movements (Eccles, 1982). This hypothesis was unchallenged for several decades, until an fMRI experiment demonstrated that neural activities in the frontal and parietal cortices encode outcomes of free decisions up to ten seconds before the subject registers awareness of those decisions (Soon et al., 2008). The present experiment extends these findings by showing that the neural precursors of self-initiated movement, as indexed by readiness BOLD signals, are widely distributed over all four lobes of the brain, including the sensory, motor, and association cortices. Endogenous accumulation of neural activities in networks of multiple cortical regions precedes generation of self-initiated movement. Because the evidence is correlational, further research is necessary to clarify whether the accumulation represents a cause of self-initiated movement.

Substantial readiness BOLD signals were observed in the visual and auditory cortices, even absent sensory stimulation. Retrospective examinations of activation maps in previously published experiments revealed occipital activations associated with free decision making (Rowe et al., 2010; Zhang et al., 2012), which provide further support for our results. The neural mechanisms responsible for the buildup of neural activities in visual and auditory areas require clarification, but subjects’ continued awareness that they had to move their hand could have biased the stochastic firings of sensory cortical neurons to accumulate over time, similar to how attention boosts evidence accumulation (Krajbich and Rangel, 2011). Whatever the mechanism, once the ramping neural activities in the visual and auditory cortices reach a threshold, they would trigger a series of neural events in connected brain regions, in a manner similar to how externally induced neural activities trigger subsequent neural events. Consistent with this hypothesis, neural processes in the SMA and frontoparietal cortices, that support endogenously generated actions, are virtually indistinguishable from those supporting externally generated actions (Hughes et al., 2011; Wisniewski et al., 2016).

The observation of readiness BOLD signals in the SMA was an expected finding that confirmed previous results (Weilke et al., 2001; Cunnington et al., 2002; Soon et al., 2008). This corroborates the role of the SMA in the generation of self-initiated movements. However, it is questionable if the SMA serves as the ultimate origin of self-initiated movements, as was previously believed (Eccles, 1982). The earliest information that predicts the outcomes of free decisions is encoded in frontal and parietal cortices, rather than in the SMA (Soon et al., 2008). Additionally, premovement activation within SMA (similar to our readiness BOLD signals) can be recorded when a delay period of several seconds or more is inserted between an external visual cue and movement execution (Hanakawa et al., 2008; Kasess et al., 2008). Therefore, the SMA is involved in the preparation of movements, respective of whether the movement is triggered externally or internally.

The rest of brain regions that exhibited readiness BOLD signals (i.e., precuneus, right IPL, right IFG, and insula) are known to contribute to self-initiated movement (Jenkins et al., 2000; Soon et al., 2008; Haggard, 2009; Hoffstaedter et al., 2013), but did not previously display evidence for early buildup of neural activities during the premovement period. The precuneus and the IPL are major nodes of the default mode network (DMN; Raichle et al., 2001; Buckner et al., 2008; Raichle, 2015), and spontaneous neural activities in DMN contribute to the generation of internally generated movements (Goldberg et al., 2008; Soon et al., 2013). A low-intensity electrical stimulation of the IPL generates intent to move while high-intensity stimulation produces belief of movement performance (Desmurget and Sirigu, 2009; Desmurget et al., 2009; Haggard, 2009). Decisions during a free-timing task were predicted from the spatial pattern of activation in the parietal cortex, including the precuneus, several seconds before movement onset (Soon et al., 2008, 2013). The right dominance in IPL is consistent with previous reports (Jenkins et al., 2000; Hoffstaedter et al., 2013), and insula has been suggested evaluate the outcomes of intentional action decisions (Brass and Haggard, 2010). The right IFG is involved in endogenous inhibition of action (Aron et al., 2014). These areas likely contribute to the self-initiation of movement concerning higher stages of neural processing that link neural activities in sensory cortices and SMA.

Subjects’ movements in this experiment were spontaneously generated without an external trigger, pointing to a hypothesis that one or more resting-state networks (RSNs) contributed to the self-initiation of movement. To our knowledge, there is no single known RSN that contains all brain regions that exhibited readiness BOLD signals in our experiment (Cole et al., 2010; van den Heuvel et al., 2010; Barkhof et al., 2014). Rather, the delineated regions represent major nodes of several different RSNs, namely the sensorimotor, default mode, frontoparietal, salience, visual, and auditory networks (Smith et al., 2009; Cole et al., 2010; Spreng et al., 2013; Barkhof et al., 2014; Raichle, 2015). Multiple RSNs might interact and contribute to the generation of self-initiated movement, which is a hypothesis warranting further investigation. Functional interaction between RSNs is a topic of ongoing research (Spreng et al., 2013; Zalesky et al., 2014; Spadone et al., 2015).

Our unique experimental design permitted the discovery of novel findings. Several previous EEG and fMRI studies have investigated the neural substrates of self-initiated movements, but most only used short time windows (typically <5 s) for analyzing premovement neural activities (Ball et al., 1999; Haggard and Eimer, 1999; Weilke et al., 2001; Cunnington et al., 2002, 2003; Matsuhashi and Hallett, 2008), whereas our premovement time window was 15 s. In contrast, Soon et al. (2008) used a long premovement time window of 10 s and revealed slowly increasing neural activities in the SMA, but not in the other regions identified in the current study. A possible reason for this discrepancy was that Soon et al. (2008) inherited the experimental paradigm of Libet (1985) and used updating visual stimuli to mark the timing of subjective decisions. Such external sensory stimulation would disturb spontaneous neural activities in the visual cortex as well as in other brain areas that were functionally connected with the visual cortex. The fixed visual stimulus in our paradigm was more suitable for observing internally driven neural activities without external confounds.

To summarize, self-initiated movements are preceded by slowly increasing neural activities in widely distributed cortical regions throughout the sensory, motor and association cortices. Considering that spontaneous neural activities in the “resting” brain are organized in the same functional networks as those that support various motor and cognitive tasks (Smith et al., 2009), it is plausible that shared neural mechanisms underlie self-initiated movements and externally-triggered movements, not just during motor processing throughout the final stages of movement execution (Hughes et al., 2011), but also during the intermediate stages of neural processing in the frontoparietal cortices (Wisniewski et al., 2016) and, moreover, the initial input stages in the sensory cortices. In this view, the critical difference between self-initiated versus externally-triggered movements is that whether the accumulation of evidence in sensory cortices is driven internally from stochastic firings of neurons, or it is triggered externally by sensory inputs. Further studies will likely test this novel hypothesis and clarify how multiple cortical regions interact during the premovement period to generate behaviors characterized as being based on free will.

## Synthesis

Reviewing Editor: Christophe Bernard, INSERM & Institut de Neurosciences des Systèmes Decisions are customarily a result of the Reviewing Editor and the peer reviewers coming together and discussing their recommendations until a consensus is reached. When revisions are invited, a fact-based synthesis statement explaining their decision and outlining what is needed to prepare a revision will be listed below. The following reviewer(s) agreed to reveal their identity: Aaron Schurger.

Both reviewers and myself agree that the neural origins of self-initiated movement is an important topic. Because your results point to an alternative (complementary?) mechanism to current thinking, we believe that the demonstration must be strengthened, as we found several issues that need to be fixed. No additional experiment is necessary, but extreme care should be devoted to statistics (you may consider seeking help from an statistics expert), as well as data analysis and interpretation.

Statistics and methods:

In the methods section on page 7, you state “First, we created whole-brain activation maps for identifying candidate regions of interest (ROIs) that potentially exhibited readiness BOLD signals.” You do not explain how they did this, and this needs to be clarified. We assume this was done with a simple contrast, but it should be spelled out in full detail. One potential problem is that if you did the statistical analyses on the regions identified with another statistical analysis (creation of uncorrected T-maps) then this might constitute cherry picking (double dipping / non-independence) of effects of interest. What kind of cross-validation did you perform? If none was necessary then you need to explain why. In any case, the fMRI data analysis methods need to be spelled out in more detail.

We also wonder about the possible need for a correction for multiple comparisons, since you identified several regions and performed t-tests on all of them. You need to specify whether or not they corrected for multiple comparisons, and if not justify why it was not necessary to do so. This part of the data analysis must be very clearly explained.

So in summary, the methods need to be spelled out in more detail and you have to address the issue of multiple comparisons.

Data interpretation:

We already have ample evidence of early precursors of self-initiated movement. So the novel finding here is that these early precursors can be found in several brain areas that had not been previously identified. That is in fact novel, but you may provide a better explanation why this particular result is important. One compelling possibility may be that early precursors to self-initiated movement might be common to a functional “resting-state” network, that includes, of course, the SMA. It would be particularly interesting if (for example) you could show that the set of regions that you identified all belong to a previously identified functional resting-state network . One piece of obscure prior evidence that you could cite and comment on is the finding of a slow buildup of activity in the temporal lobe preceding self-initiated movements (Fried et al, 2011, supplementary material). Little mention was made of this finding, perhaps because it is puzzling to explain. But if the SMA and this part of the temporal lobe both belong to the same functional network, then that might explain the shared buildup in those two regions. The importance of you results may be conveyed more clearly to the reader.

You refer to the BOLD signal beginning 10 seconds prior to self-initiated movement as the BOLD correlates of the readiness potential seen on EEG. Is this an accurate statement? The readiness potential/Bereitschaftspotential as measured on EEG (a much more temporally accurate measure than fMRI) appears ~1 second prior to movement initiation. While there may well be slow accumulation of neural (BOLD) activity prior to self-initiated movement, this does not necessarily equate to the BOLD equivalent of the readiness potential. Could you clarify?

You report that several areas are preferentially activated in the free-timing pre-movement period. This result is unclear from the activation map shown in figure 2, given that a clear signal is only readily apparent at T=0 timepoint. Figure 2 would also benefit from having an anatomical brain as underlay.

Discussion:

The question arises of why prior fMRI studies have not found a readiness buildup in areas other than the SMA. You suggest that this was probably due to the use of rapidly updating visual stimuli used to mark the time of subjective decisions. However, the you do not explain WHY the use of rapidly updating visual stimuli would tend to mask the kind of effects that you found. It is not clear why rapidly updating visual stimuli would obscure the slow buildup in areas other than the SMA. This should be explained more clearly.

The novelty of the findings may be a bit overstated, particularly the detection of an early “readiness BOLD signal” beginning as early as 10 seconds prior to motor execution. It is not clear that you have demonstrated that this signal begins 10 seconds prior to movement execution. In addition, as you mention, Soon et al (2008) have previously demonstrated an fMRI pre-movement correlate beginning approximately 8 seconds prior to movement initiation.

Minor

Why did you only recruit males? This should be explained, and included as a limitation.

The fMRI images were acquired with slice thickness of 5mm, and only 15 axial slices were collected to cover the whole brain. You also used a high degree of smoothing during image processing (8-mm FWHM). This lower resolution image acquisition should be included as a limitation.
